# Minimally Invasive Laparoscopic Donor Nephrectomy With a Pfannenstiel Incision Using Size-Reduced Trocars

**DOI:** 10.7759/cureus.67763

**Published:** 2024-08-25

**Authors:** Jun Hagiuda, Tsukasa Masuda, Ryohei Takahashi, Satoshi Tamaki, Ken Nakagawa

**Affiliations:** 1 Urology, Tokyo Dental College, Ichikawa General Hospital, Chiba, JPN

**Keywords:** minimally invasive laparoscopy, size-reduced trocar, pfannenstiel, laparoscopic donor nephrectomy, cosmesis

## Abstract

Background: Laparoendoscopic single-site surgery is performed during laparoscopic donor nephrectomy (LDN) to reduce donor invasiveness. However, the procedure is difficult and does not improve cosmesis when the incision is made at the umbilicus. Therefore, we proposed a minimally invasive LDN with a Pfannenstiel incision using size-reduced trocars (mLDN) to achieve cosmesis and operability and aimed to assess its efficacy and safety.

Methods: A total of 27 patients who underwent mLDN were recruited. Data on estimated blood loss, operative time, pneumoperitoneum time, warm ischemic time (WIT), complication rate, non-steroidal anti-inflammatory drugs (NSAIDs) used, and recipient serum creatinine levels were collected retrospectively. In mLDN, the Pfannenstiel position was incised to approximately 6 cm to retrieve the kidney, and three size-reduced trocars were placed in the left upper abdomen (2.5 mm and 5 mm) and umbilicus (5 mm).

Results: The median operation time and pneumoperitoneum time were 245 and 194 minutes, respectively. The median WIT was 276 seconds, and the serum creatinine levels of the recipients at seven days and one, three, six, and 12 months were significantly improved compared with baseline. No intra- and postoperative complications (Clavien-Dindo grade ≥ 2) were observed, and no patients used additional NSAIDs after the operation. The scarring in the mLDN group was unnoticeable postoperatively.

Conclusions: mLDN can be performed safely, with high cosmesis, and with operability similar to that of conventional LDN. Although the WIT tended to be long, the function of the harvested kidney was maintained, and the use of analgesic NSAIDs was lower in this procedure. Our procedure should be considered as an option for LDN.

## Introduction

Laparoscopic donor nephrectomy (LDN) is an effective treatment for kidney transplantation compared to open donor nephrectomy in terms of early recovery and less pain, with similar complication rates and graft function. In 2020, >95% of donor nephrectomies in Japan were performed under laparoscopic surgery [1]. Laparoendoscopic single-site (LESS) donor nephrectomy was recently introduced to make the procedure less invasive for healthy donors. Several randomized controlled studies have also confirmed its non-inferiority compared with conventional LDN, which can be attributed to the lower number of trocars and shorter wound length, improving the pain scores in the LESS groups [2-6]. However, cosmetic scores were similar between the LESS and conventional LDN groups. One reason for this may be that LESS donor nephrectomy involves incision of the umbilicus, leaving a wound at the center of the body, which might disfigure the body image of the patient [2,4,6]. Additionally, Kurien et al. reported that surgeons experienced more difficulty with LESS donor nephrectomy than with LDN due to the decreased distance between each trocar during LESS surgery, resulting in the devices colliding with each other [2].

Consequently, since 2014, we have attempted several approaches to achieve minimal invasiveness, better cosmesis, and a technically easy operation for transperitoneal left donor nephrectomy. At first, we initiated LESS donor nephrectomy through an umbilical incision. However, as mentioned previously, the surgeon and scopist were uncomfortable because of the narrow space and the high level of skills required. In addition, the wound in the middle of the abdomen was painful and noticeable. Second, we moved the incision to a Pfannenstiel and inserted a SILS^TM^ port (Covidien, Tokyo, Japan) at the umbilicus. In this procedure, although the Pfannenstiel incision was hidden by pubic hair and was cosmetically better, the operability worsened because of the SILS^TM^ port. Therefore, we placed 2.5-mm and 5-mm ports in the left upper abdomen instead of the umbilical SILS^TM^ port. Thin-diameter forceps were used through a 2.5-mm trocar. Although this resulted in improved operability, the scopist still experienced difficulty because the camera had to be inserted through the Pfannenstiel incision at a distance from the harvested kidney. In addition, operative devices such as a stapler or clip to ligate the renal artery and vein had to be inserted from the GelPOINT® device (Applied Medical, Rancho Santa Margarita, California) installed at the Pfannenstiel wound and often collided with the scope. In a final attempt, we inserted an additional 5-mm trocar from the umbilicus for the scope. This method resulted in similar operability to that of conventional laparoscopic nephrectomy because the positions of the three trocars (camera and surgeon's both hands) formed a triangulation. Moreover, the wound of the additional 5-mm port placed at the umbilicus position and a 2.5-mm trocar became less noticeable postoperatively.

Another advantage of Pfannenstiel wounds is that devices for clipping renal vessels approach the renal hilum vertically. Therefore, we can harvest kidneys with long arteries and veins, which are beneficial for anastomosis. Accordingly, in the present study, we aimed to analyze the outcomes of LDN with a Pfannenstiel incision using size-reduced trocars and elucidate the efficacy and safety of our novel procedure.

## Materials and methods

Since 2017, 27 patients who underwent minimally invasive LDN with a Pfannenstiel incision using size-reduced trocars (mLDN) at the Tokyo Dental College Ichikawa General Hospital were enrolled in this study. In all cases, the left kidney was harvested using a transperitoneal approach. This study was approved by the Ichikawa General Hospital Scientific Council and ethics committee (I 22-18). Written informed consent was obtained from all patients before surgery.

Preoperative patient data, including age, sex, height, weight, body mass index, serum creatinine level, and 24-hour creatinine clearance, were collected. In addition, we obtained the following intra- and postoperative patient data: estimated blood loss, operative time, pneumoperitoneum time, warm ischemic time (WIT), time of hospital stay, time to oral intake, complication rate, number of analgesic agents (non-steroidal anti-inflammatory drugs (NSAIDs)) used, and serum creatinine levels of the donor at one month. The serum creatinine level of the recipient was followed up at one week and one, three, six, and 12 months.

Surgical procedure

**Figure 1:**
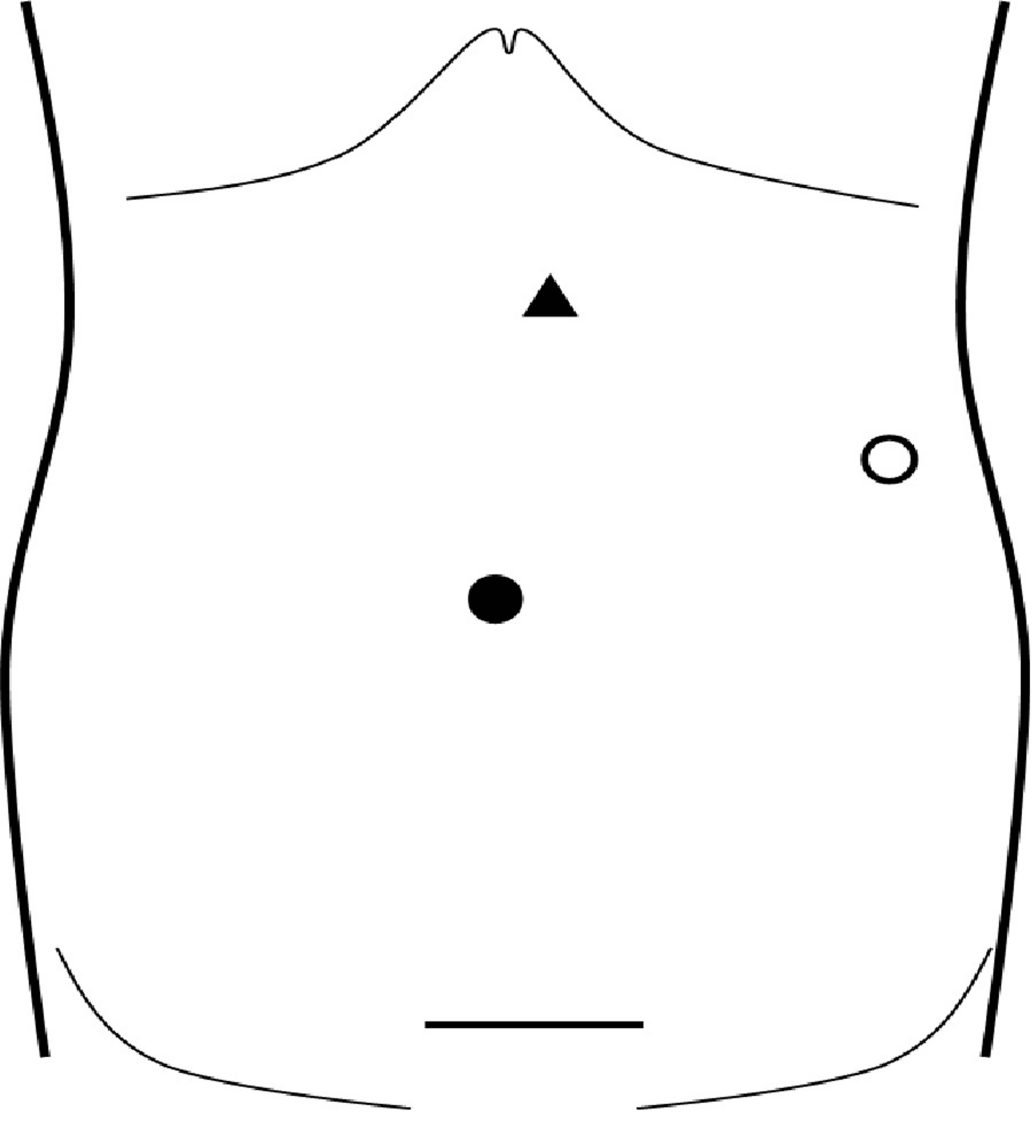
Pfannenstiel wound and trocar configuration ○: 5-mm trocar for the right hand; ●: 5-mm trocar for the camera inserted from the umbilicus; ▲: 2.5-mm trocar for thin-diameter forceps.

The surgeries were performed by JH and KN, who are experts in laparoscopic surgery. Patients were placed in the flank position. Initially, the Pfannenstiel site was incised, approximately 6 cm, and the rectus abdominis was carefully divided to avoid cutting it off and covered by a GelPOINT® device. After creating a pneumoperitoneum, two 5-mm trocars were inserted at the umbilicus for the flexible camera (EndoEye®, Olympus Corporation, Tokyo, Japan) and lateral to the left upper abdomen for the operating devices (Opti4 laparoscopic electrode® (Covidien, Tokyo, Japan) and LigaSure^TM^ Maryland (Covidien Japan, Tokyo, Japan)). In addition, a 2.5-mm trocar was placed in the upper left abdomen for the Endo Relief forceps (Hope Denshi Co., Ltd., Chiba, Japan) (Figure 1).

**Figure 2:**
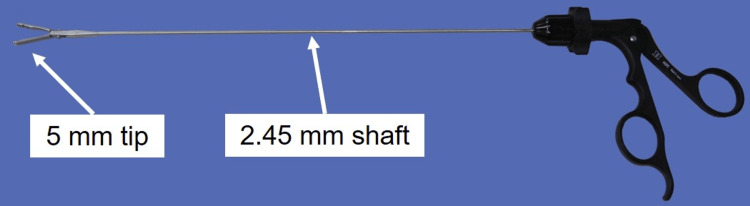
Thin-diameter forceps used in our procedure The Endo Relief forceps comprised a 5-mm tip, a 2.45-mm shaft, and a handpiece.

The forceps were assembled by docking the 5-mm tip with a 2.45-mm shaft inserted from a 5-mm trocar and a handpiece prepared outside the 2.5-mm trocar (Figure 2).

After trocar insertion, the descending colon, pancreas, and spleen were mobilized, and the renal hilum was exposed. The adrenal and gonadal veins were divided using a titanium clip and LigaSure^TM^. Following the division of the ureter, the kidney was prebagged, and the renal artery and vein were ligated by a stapler (Signia^TM^, Covidien, Tokyo, Japan) and clip (Hem-o-lok®, Teleflex, North Carolina), respectively. The graft was extracted from the Pfannenstiel incision via the GelPOINT® device.

Statistical analysis

Statistical differences in the serum creatinine levels of the recipients at seven days and one, three, six, and 12 months compared with baseline were determined using a paired t*-*test. A p-value of ≤ 0.05 was considered statistically significant.

## Results

The patient characteristics are shown in Table 1.

**Table 1:** Background characteristics and variables The results were expressed as means ± standard deviations.

Characteristics	mLDN
Cases	27
Age (years)	53.7 ± 11.4
Male vs. female	11 vs. 16
Height (cm)	161.6 ± 9.2
Weight (kg)	63.2 ± 10.4
Body mass index (kg/m^2^)	24.1 ± 2.8
Serum creatinine level (mg/dL)	0.75 ± 0.16
24-hour-creatinine clearance (mL/min)	119.3 ± 24.1

The number of female donors was slightly higher than that of male donors. Intra- and postoperative data are shown in Table 2.

**Table 2:** Surgical outcomes *Results were expressed as medians (ranges). **Results were expressed as means ± standard deviations.

Parameters	mLDN
Estimated blood loss (mL)*	50 (0–450)
WIT (s)*	276 (195–478)
Operation time (min)*	245 (184–429)
Pneumoperitoneum time (min)*	194 (131–354)
Length of hospital stays (days)*	6 (4–13)
Number of non-steroid anti-inflammatory drugs used	0
Serum creatinine level of the donor at one month**	1.12 ± 0.24

All surgeries were performed without an additional trocar or open nephrectomy conversion. The median estimated blood loss was low (50 mL), and no patient underwent a blood transfusion. All patients began oral intake and ambulation the following day. Clavien-Dindo ≥ grade 2 postoperative complications were not observed. In addition, no patients developed wound-related complications such as infection or incisional hernia. An epidural anesthesia catheter was inserted for approximately three days. Acetaminophen was primarily administered as an additional analgesic agent, and no patients required NSAIDs or narcotic therapy.

**Figure 3:**
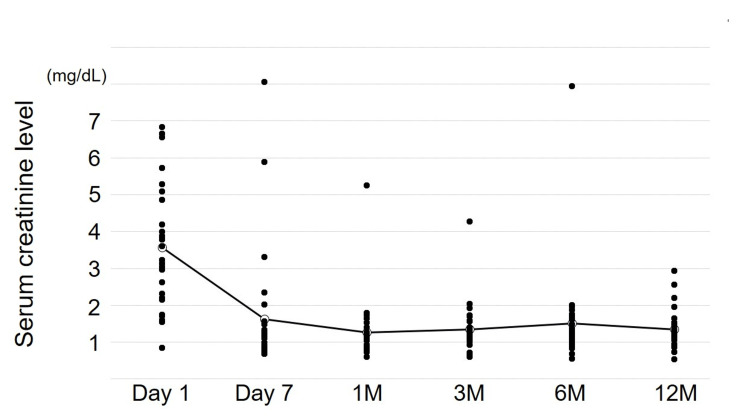
Changes in the serum creatinine levels of the recipients Serum creatinine levels improved at one week and at one, three, six, and 12 months compared to baseline.

The median operation time and pneumoperitoneum time were 245 and 194 minutes, respectively. The median WIT was 276 seconds, and the recipients' serum creatinine levels improved significantly at seven days and at one, three, six, and 12 months compared with baseline (Figure 3). Delayed graft function was not observed; however, one graft loss occurred due to hyperacute rejection.

## Discussion

In this study, we analyzed the outcomes of mLDN with a Pfannenstiel incision using size-reduced trocars and elucidated its efficacy and safety. Procedural safety and maintenance of graft function are required for donor nephrectomy. Minimally invasive procedures and cosmesis are additional factors that are expected by donors. LESS donor nephrectomy was introduced to address these points; however, the procedure is difficult, and cosmesis satisfaction may be reduced when the incision is placed near the umbilicus. Consequently, we reduced the trocar size with a configuration similar to that previously reported [6-8], wherein three trocars, including the umbilicus camera port and two other trocars in the left upper abdomen, were placed with a Pfannenstiel incision to maintain operability and improve cosmesis.

In the present study, no patient required additional trocars or open nephrectomy conversion. In previous studies, approximately 10% of LESS donor nephrectomies required additional trocars [3,4,9-11]. In some cases, an additional trocar, which was not a "single incision," was inserted at the beginning of the operation, highlighting the need for skilled surgeons to perform LESS donor nephrectomies. Similarly, Kurien et al. reported that surgeons experience significant difficulty in upper pole separation and renal pedicle dissection in LESS donor nephrectomies. An additional trocar was inserted during adrenal vein division and upper pole separation in 40% and 52% of cases, respectively [2]. The difficulty of these steps may be due to the lack of countertraction and the clashing of instruments in LESS donor nephrectomies. Conversely, in our procedure, the surgeon can perform the surgery with good maneuverability and triangulation, similar to the conventional LDN. Moreover, surgeons are able to insert additional instruments to make countertraction from GelPOINT® on the Pfannenstiel incision. We hypothesized that this better operability would improve surgical safety. We did not encounter the need for blood transfusions or any intra- and postoperative complications (Clavien-Dindo grade ≥ 2).

**Figure 4:**
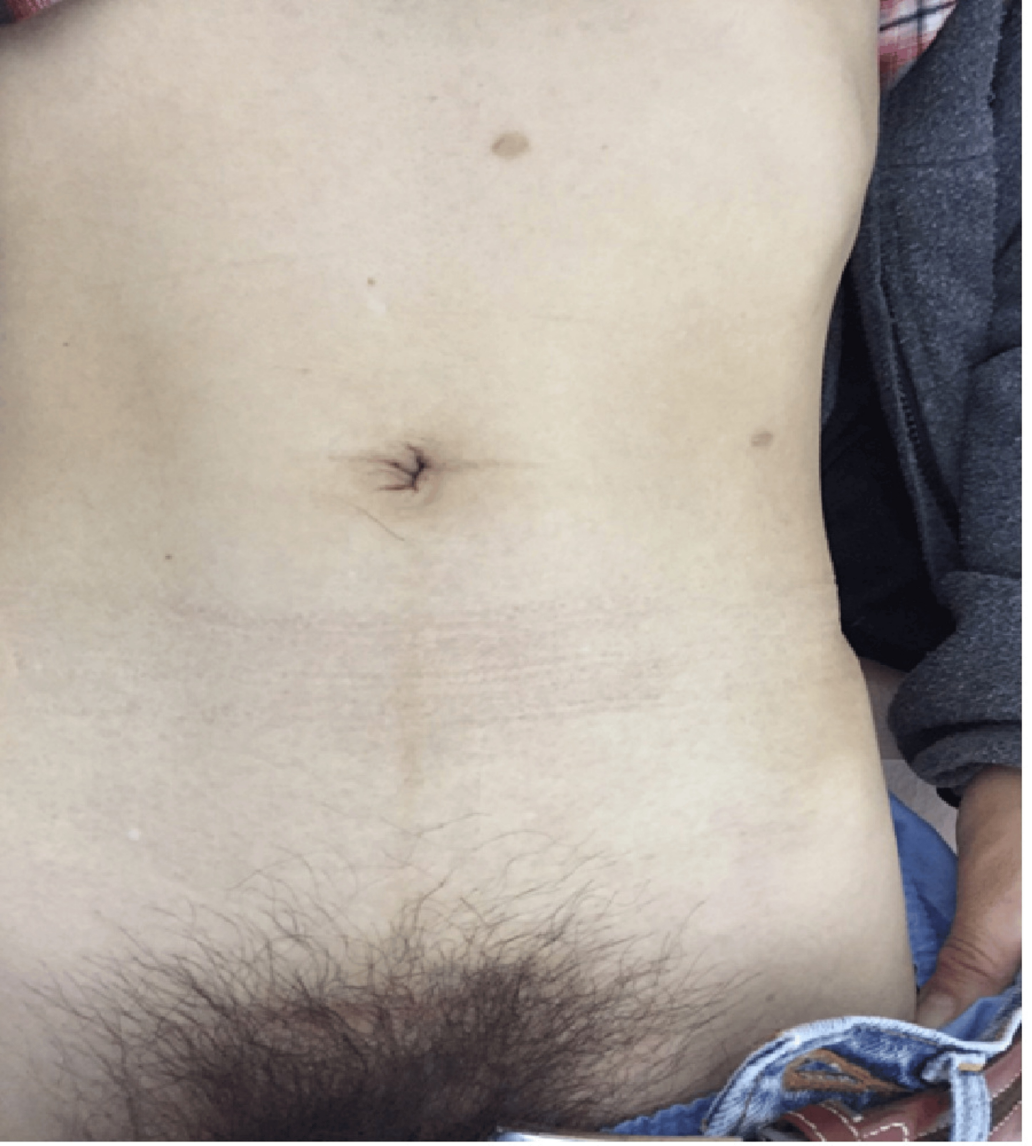
Abdominal scar in our procedure after six months The Pfannenstiel incision is hidden in the pubic hair. The scars of the 5-mm trocar at the umbilicus and the 2.5-mm trocar were unnoticeable.

Cosmesis is an important factor in these surgeries because young female patients are candidates for donors. We assumed that the size and location of the incision or number of trocars used may be related to cosmesis. Therefore, in this study, a retrieval incision was made in the Pfannenstiel position. In most cases, the wound was hidden by the patient's pubic hair. In addition, the wound of the 5-mm umbilicus trocar became invisible, and the scar of the 2.5-mm trocar was less noticeable (Figure 4).

In previous studies, patients' satisfaction with the Pfannenstiel incision was higher than that of iliac fossa or midline incisions above the umbilicus [7,12]. However, there was no difference in cosmetic scores between patients who underwent LDN and LESS donor nephrectomy with an umbilical incision [2,4,6]. This indicates that the location of the retrieval incision may affect patient satisfaction more than the number of trocars used. Therefore, we expected that the patients who underwent our procedure, particularly young female donors, would be satisfied with their cosmesis. However, further analyses of cosmetics and satisfaction are required to validate these findings.

Another advantage of the Pfannenstiel incision is the reduction in pain. At our institution, most patients received epidural catheters and did not require additional NSAIDs or narcotic agents. Several studies have compared the pain score or frequency of rescue analgesic agent usage [12-17], and in some of these studies, the Pfannenstiel incision was superior to other incisions [12,15]. Moreover, avoiding the use of narcotic agents or NSAIDs leads to early recovery from postoperative intestinal paralysis and protects donor renal function. In our study, the median number of acetaminophen administrations per day (mostly 300 mg) after epidural catheter removal was only one. We carefully approached the intraperitoneal route by splitting the rectus muscle, which helped relieve postoperative pain. In addition, we expected that trocars with a reduced size (2.5 mm) would be less painful than the 5- or 12-mm trocars.

In addition to reducing pain, the Pfannenstiel incision carries the benefit of preventing incisional hernias. A lower incidence of incisional hernias compared to other incisions in laparoscopic nephrectomy and colorectal surgery was previously reported [18,19]. Guo et al. suggested that incisional hernias could be prevented by preserving the blood supply of the rectus abdominis, related to early wound healing, formation of the right angle between the rectus abdominis sheath and muscle against internal pressure, and staggered incision directions of the skin, fascia, muscle, and peritoneum [19]. Because the occurrence of an incisional hernia will disturb the cosmesis and necessitate an additional hernia repair operation, we suppose that the lower incidence of this complication is one of the important merits of LDN.

Nevertheless, the association between retrieval incision and WIT length remains controversial. The median length of WIT in our study was 276 seconds, which was longer than that of conventional LDN performed at our hospital (data not shown). The main reason for a longer WIT may be the distance between the kidney and the Pfannenstiel incision, resulting in the longer time required to insert and remove each instrument and the harvested kidney. A similar discussion was reported in previous studies [13,16]; however, no statistical differences were observed based on the incision location in other studies [7,12]. Another reason may be the small intestine falling close to the Pfannenstiel incision due to the patient being in the flank position. Consequently, the surgeon is obligated to insert the instrument carefully to avoid injuring the intestines. Nevertheless, previous reports showed no differences in the recipients' renal function. In our study, most recipients' serum creatinine levels improved, and the serum creatinine levels at one month were similar to those of conventional LDN (data not shown). Therefore, we assumed that although WIT was prolonged, it had no negative effects on harvested kidney function. Nonetheless, long-term follow-up is required to determine the influence of our procedure on graft function because the recipients' creatinine levels will increase gradually over many years.

Despite its novelty, our study has a few limitations. First, the analysis was single-arm, and the sample size was small. In addition, we did not evaluate cosmesis or patient satisfaction. Second, the follow-up period was relatively short. Therefore, long-term follow-ups are required to determine the efficacy of the procedure.

## Conclusions

LDN with a Pfannenstiel incision using size-reduced trocars can be safely performed with better cosmesis and operability, similar to conventional LDN. Although WIT tended to be long, the function of the harvested kidney was similar, and analgesic NSAID use was lower in this procedure. Currently, we believe that our procedure may be considered one of the best treatment options for LDN, with well-balanced operability, invasiveness, and cosmesis.
